# Preclinical Toxicity and Immunogenicity of a COVID-19 Vaccine (ZF2001) in Cynomolgus Monkeys

**DOI:** 10.3390/vaccines10122080

**Published:** 2022-12-05

**Authors:** Hongzhong Yang, Wei Pan, Guoyu Chen, Enqi Huang, Qijiong Lu, Yunxiang Chen, Ying Chen, Zhengbiao Yang, Lei Wen, Siming Zhang, Cong Xu, Wanqiang Lv, Lianpan Dai, Changwei Wu, Lijiang Zhang

**Affiliations:** 1 Center of Safety Evaluation and Research, Hangzhou Medical College, Hangzhou 310053, China; 2 Key Laboratory of Drug Safety Evaluation and Research of Zhejiang Province, Hangzhou Medical College (Zhejiang Academy of Medical Sciences), Hangzhou 310053, China; 3 Anhui Zhifei Longcom Biopharmaceutical Co., Ltd., Hefei 230088, China; 4 CAS Key Laboratory of Pathogen Microbiology and Immunology, Institute of Microbiology, Chinese Academy of Sciences, Beijing 100101, China

**Keywords:** COVID-19, ZF2001 vaccine, safety evaluation, immunogenicity, cynomolgus monkey

## Abstract

Although the new coronavirus disease 2019 (COVID-19) outbreak occurred in late 2019, it is still endemic worldwide, and has become a global public health problem. Vaccination against SARS-CoV-2 is considered to be the most effective intervention to prevent the spread of COVID-19. ZF2001 is a recombinant protein vaccine based on SARS-CoV-2 receptor-binding domain (RBD) subunit which contains aluminum adjuvant. In order to advance our research on ZF2001 into clinical trial, we investigated the general toxicity and immunogenicity of ZF2001 in cynomolgus monkeys and assessed the possible target organs for vaccine-induced toxicity. In the present research, we observed no significant systemic toxicities and abnormal cardiovascular and respiratory events following four times injections of intramuscular ZF2001 in cynomolgus monkeys. Histological examination revealed recoverable inflammatory changes in quadricep muscle and adjacent lymph node at the vaccine injection site. As expected, the vaccine can produce a strongly specific binding antibody and neutralizing antibodies in cynomolgus monkeys after inoculation. Taken together, our regulatory toxicology research proves the safety and immunogenicity of the ZF2001 vaccine, supporting its entry into large scale clinical trials.

## 1. Introduction

Coronavirus disease 2019 (COVID-19) is a kind of pneumonia caused by novel coronavirus infection. In severe cases, COVID-19 can cause severe acute respiratory syndrome, renal failure, and even death. According to the latest statistics of the World Health Organization, there have been over 520 million confirmed cases globally, with over 6.28 million deaths due to COVID-19 worldwide [1]. The number of individuals infected with COVID-19 is still growing at present. Many epidemiological studies have reported that COVID-19 has an important influence on neurological, respiratory sequelae and invasive pulmonary aspergillosis (IPA) [2,3]. At the early epidemic stage in 2020, there was a shortage of effective drugs for the prevention and treatment of novel coronavirus. The research and development of vaccines preventing COVID-19 is urgent.

The Severe Acute Respiratory Syndrome Coronavirus 2 (SARS-CoV-2) was first isolated and characterized by the National Pathogen Resource Center, Chinese Center for Disease Control and Prevention (CDC) [4]. Coronaviruses are positive-sense, single-stranded RNA viruses enveloped by a lipid membrane in which the structural proteins S, M and E are inserted. The SARS-CoV-2 virus contains four structural proteins, including spike (S), envelope (E), membrane (M), and nucleocapsid (N) proteins. The S1 subunit of SARS-CoV-2 virus spike protein possesses a receptor-binding domain (RBD), which binds to angiotensin-converting enzyme 2 (ACE2) of the host cell membrane [5]. The virus releases its genomic RNA into the cytoplasm for reverse transcription and gene expression, resulting in infection of the host cell. Therefore, the RBD of viral S1 protein is the most targeted antigen in the development of a vaccine against SARS-CoV-2 [6,7].

**Figure 1:**
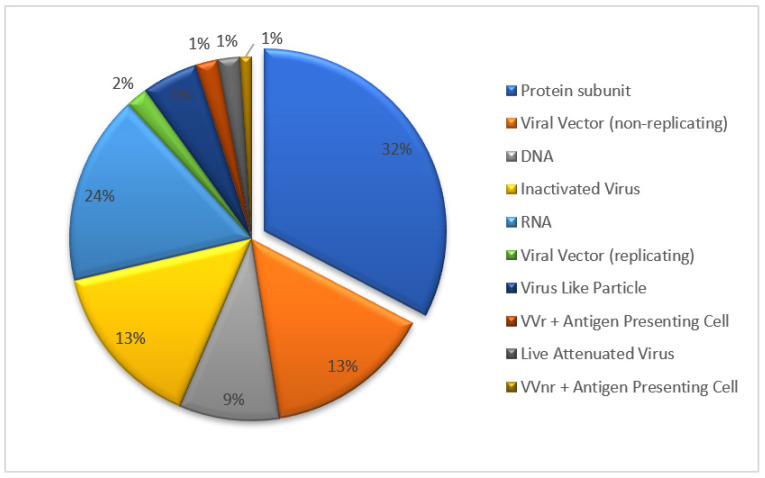
The types and proportions of COVID-19 vaccine undergoing clinical trial.

Several types of vaccines have been developed against SARS-CoV-2, including inactivated vaccines, live-attenuated vaccines, viral vector vaccines, recombinant subunit vaccines and genetic-based vaccines (mRNA and DNA) [8]. The recombinant protein vaccine forms the largest proportion of candidate vaccines undergoing clinical trial (Figure 1, update to 18 November 2022). The recombinant protein vaccine was obtained through several steps: the gene cloning of pathogen-specific antigen, incorporation into expression systems such as Chinese hamster ovary (CHO) cells, and in vitro antigen-specific protein cultivation in a large scale. The recombinant vaccine benefits more safety profile from pure antigen ingredient, without introduction of pathogen gene, no gene replication risk in vivo, in addition to the convenience of scale-up production and storage (2–8 °C). Taking the R319 to K537 sequence chain in the RBD domain of SARS-CoV-2 as a key bone, two chains were connected by a disulfide bond to obtain the ZF2001 key antigen structure. This design strengthened the ZF2001 vaccine’s thermal stability and RBD binding affinity [9]. The efficacy study of ZF2001 has shown that the vaccine can protect mice and rhesus monkeys against SARS-CoV-2 challenge [10]. The ZF2001 vaccine targeting the RBD region of the SARS-CoV-2 structure remains its protective capacity against SARS-CoV-2 circulation variants such as delta, gamma and omicron [11].

Before the new vaccine is approved for first-in-human research, preclinical safety assessment studies of the vaccine are required to reduce the risk of vaccine-testees. The ZF2001 vaccine was constructed with the SARS-CoV-2 receptor binding domain (RBD) tandem dimer as its specific antigen, which may bring unknown risks to the recipient. A lack of safety data blocks its use in clinical trial. It is necessary to declare the unknown toxic reaction through preclinical safety assessment studies especially repeat-dose toxicity studies in cynomolgus monkeys. In order to support ZF2001’s successful entry into clinical study and obtain the licensure of the vaccine as soon as possible, shortly after the COVID-19 epidemic in 2020, our laboratory started a full set of preclinical safety evaluation studies of ZF2001. Here, we present the comprehensive repeat-dose toxicity study, including safety pharmacology and immunogenicity in cynomolgus monkeys as part of the preclinical safety study portfolio.

## 2. Materials and Methods

### 2.1. Study Design

A total of 40 cynomolgus macaques aged from 3.0 to 3.5 years and weighing from 2.95 to 3.67 kg, half male and half female, were purchased from JinGang Biotechnology Co., Ltd. (Hainan, China), China (Laboratory Animal Production License No: SCXK (Qiong) 2015-0001, Laboratory animal quality certificate No: 460001200000380). Animals were included in the study after a quarantine procedure. The animals were raised in single stainless-steel cages in ventilated rooms with filtered, non-recirculated air at a rate of 10–12 changes/h. The room was maintained under a 12:12 light-to-dark cycle and ambient temperature at 19.0–24.0 °C, humidity 57–69%. The animals were fed ad libitum and supplemented with fresh fruit daily. The animals were identified by chest tattoos number. All the animal experimental protocols and welfare complied with the related ethics regulations. This study was approved by the Institutional Animal Care and Use Committee (IACUC) of the Hangzhou medical college Animal Experiments Ethics Committee (Permit Number: GLP-2020-048). The study facility was fully AAALAC accredited (AAALAC, 2019). The study was conducted in accordance with Good Laboratory Practices (GLP).

**Figure 2:**
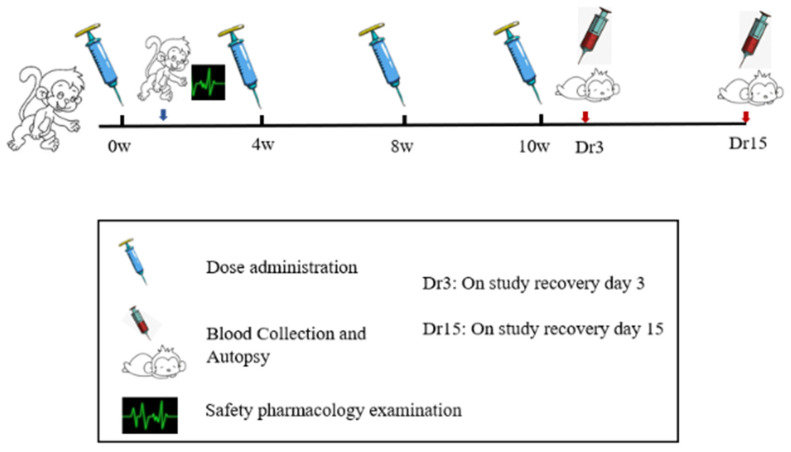
The study flowchart for safety evaluation of the ZF2001 vaccine.

ZF2001 vaccine was developed by Anhui Zhifei biological company. Each bottle of vaccine injection contained 50 μg of novel coronavirus spike glycoprotein-receptor binding domain (NCP-RBD). ZF2001 candidate vaccines were stored at from 2 to 8 °C. After 2 weeks of adaptive feeding, cynomolgus monkeys were randomized into four groups (*n* = 10), as follows: blank control (I), adjuvant control, aluminum hydroxide as an adjuvant (II), low-dose and high-dose group (III–IV). The animal number was determined in accordance with National Medical Products Administration (NMPA, China) repeat-toxicology study requirements on the number of animals and 3R principle. Groups I–II received 1.0 mL/doses saline and the adjuvant alone, which were RBD-antigen-free. Groups III and IV were injected with dosage of ZF2001 50 μg/dose and 100 μg/dose into the quadriceps muscle of the hind legs. According to the preclinical guidelines for prophylactic vaccine assessment, monkeys were vaccinated at baseline (0 W) and weeks 4 (4 W), and 8 (8 W), and 10 (10 W) for a total of four immunizations, one more dosing than the planned clinical immunization doses (N + 1 principle) [12]. After four administrations of ZF2001, 2 weeks of treatment-free recovery period followed, to judge the reversibility of vaccine toxicity if this occurred. In order to accelerate the development of the COVID-19 vaccine, the 4-week interval after the 4th dosing was shortened to 2 weeks. A flowchart of the present study is presented in Figure 2.

### 2.2. Clinical Examination

Body weight was recorded every week. The body temperature was measured a total of four times for each dosing, 0 h, 4–6 h, and on the 2nd day and 3rd day after administration, and once a week on no-dosing weeks. Hematology, blood biochemistry, electrocardiogram, serum immunoglobulin (IgG, IgM), complement (C3, C4), peripheral blood lymphocyte (PBLC) subtype (CD3+, CD4+, CD8+), ophthalmology and urine examination were performed at the end of the drug phase (10 W) and recovery period (12 W), respectively. Six and four monkeys (half male and half female) were sacrificed at the end of the dosing phase and recovery period (Dr3, Dr15), respectively. All major organs (brain, heart, kidney, liver, spleen, thymus, lung, etc.), quadricep muscle and inguinal lymph node were fixed in 4% paraformaldehyde and prepared for histological examination.

### 2.3. Immunogenicity Study

Serum samples for immunogenicity were collected from the adjuvant control group, and low and high dose groups prior to 1st dosing and 4, 4, 2 and 2 weeks after the 1st, 2nd, 3rd and 4th dosing, respectively. The SARS-CoV-2 pseudovirus preparation and neutralization assay were carried out following pre-established protocols released by the the China Food and Drug Verification Research Institute with minor modifications [13]. In brief, the plasmids of pNL4-3.luc.RE and pCAGGS-S (encoding for full-length S protein of SARS-CoV-2) were co-transfected into 293T cells. After 48 h, the supernatant containing pseudovirus was harvested, centrifuged and filtered through a 0.45 μM sterilized membrane. Coronavirus pseudovirus was provided from the China Food and Drug Verification Research Institute. The serum samples were inactivated at 56 °C, serially diluted two-fold and incubated with an equal volume of 100 TCID50 pseudovirus at 37 °C. Next, the mixture was added to pre-plated Huh7 cell monolayers in 96-well plates. After incubation for 20–28 h, the relative luciferase activity in cell extracts was measured by using Luciferase Assay System (Promega, Madison, Wisconsin USA). The neutralizing antibody (NTAb) titers are expressed as the reciprocals of the highest dilutions capable of inhibiting 50% of the CPE. A cell control, virus control and serum positive control were included in each experiment.

### 2.4. Safety Pharmacology Study

We systematically evaluated the vaccination’s effects on the cardiovascular, body temperature and respiratory systems. The former eight monkeys of each group were monitored using an EMKA non-invasive physiological signal telemetry system (EMKA, Loire, France) for 24 h after the 1st dosing. The animal number was determined in accordance with the NMPA requirements of safety study and 3R principle. The monkeys were dressed in a special vest with instrument-bearing pockets out of their reach. The animals were unrestrained, freely mobile, and fed ad libitum. The instrument collected and transmitted telemetry physiological signals, which could be distantly received by the host computer. Monkey electrocardiogram (ECG), respiration and body temperature parameters were continuously recorded for 1 h before dosing until 24 h after dosing. The hemodynamic data (systolic, diastolic, and mean arterial pressure) were measured by cuff on forelimb, for the reason of cuff loosening on freely move animals, it only can be measured on several time points under restraint control at 1 h pre-dose, 0–1, 2–3, 5–6, and 23–24 h post dose.

### 2.5. Statistical Analysis

In the present study, we used the mean ± standard deviation (SD) to describe quantitative traits, including the weight and growth gain rate, body temperature, hematology, biochemistry, electrocardiogram, immunology, organ weight and ratio and safety pharmacology measurements. The data were analyzed for statistical significance by the ANOVA test using SPSS (version 17.0, New York, USA). In the present study, we used the One-Way ANOVA test to analyze the data when comparing between multiple groups. The groups were compared using the LSD test if Levene’s test was not significant or Games-Howell test if it was significant. Differences of *p*  ≤  0.05 were considered significant in the present study.

## 3. Results

### 3.1. General Toxicity Results

**Figure 3:**
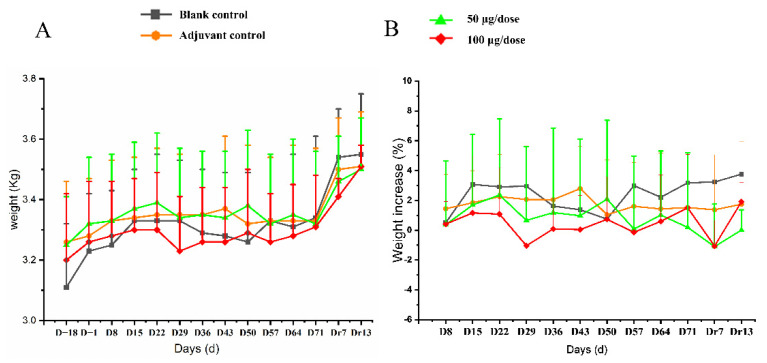
The changes of body (**A**) weight and (**B**) rate of body weight gain in cynomolgus monkeys for 4 times re-peated injections of ZF2001 vaccine study. n = 10 on study D−18-D71, *n* = 4 on study Dr7 and Dr13 in each group vs. the blank group (*p* > 0.05).

All 10 monkeys in every group exhibited no abnormal behaviors and no injection-site irritation during the experiment. No significant difference was observed in body weight or rate of weight gain between different groups (Figure 3, Table S1).

The changes in two types of leukocytes, including the increase in neutrophiles and the decrease in lymphocytes in both the adjuvant and vaccine groups, were detected at the end of the dosing phase (*p* < 0.05, *p* < 0.01). However, these changes were within the normal range of reference values, established during the acclimation period. Thus, they were not considered to be of toxicological significance. These changes in leucocyte were consistent with aluminum-adjuvants intervention [14]. No significant differences in hematology were observed at the end of the recovery period (Table 1).

Two parameters of clinical chemistry including triglyceride and albumin in vaccine-treated monkeys, were significantly higher than those in the blank control group (*p* < 0.01). These minor fluctuations remained within the normal ranges of reference values. Therefore, they are considered to be of no toxicological significance (Table 2).

We found that there were no abnormal changes in immunoglobulins, complements C3, C4 and frequencies of CD3+, CD4+, CD8+ T-cell subpopulation in the adjuvant control group and vaccine group (Table 3). Several significant temperature changes were observed on ZF2001 vaccine-treated groups compared with the blank control group on D16, D29, D31, D44 during the 2nd, 3rd and 4th dosing period (*p* < 0.05, *p* < 0.01). The mean temperature in all groups of animals slightly fluctuated over a range of 37.0–37.8 °C, which fell within our previously established reference ranges (mean 37.4, range 36.5–38.3 °C). Taken together, the vaccine had no significant influence on body temperature (Table S2).

No measures of ECG examination were markedly different from the blank control group, except that P-wave duration was longer in the high-dose group at the end of dosing phase compared to the blank group (Table S3). The ophthalmic examinations and urinalysis showed no abnormal findings.

The organ weight and organ ratio change in cynomolgus monkeys were examined at the end of the dosing phase and recovery period. No statistical differences were observed between groups (Table S4). Macroscopic observations revealed grey deposits between muscle fibers at the injection site. Some muscles presented a slightly tough texture, and hyperemia and edema appeared in adjuvant control, low-dose and high-dose groups. We found no gross pathological changes in common organs, including the heart, liver, lung, kidney and spleen, etc.

**Figure 4:**
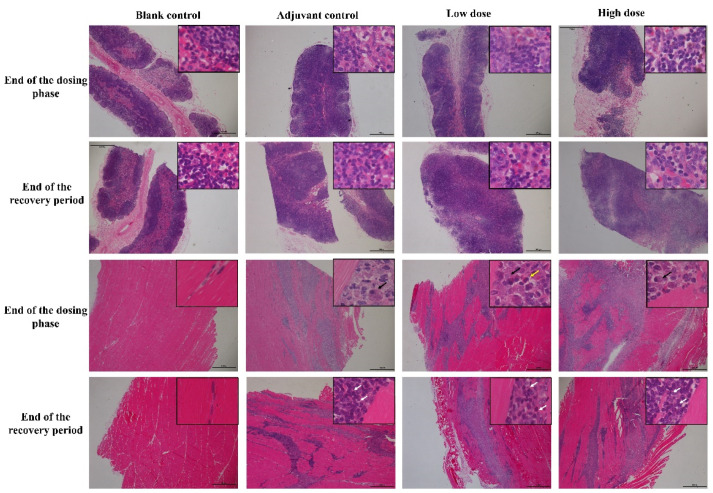
The histopathology changes of draining lymph nodes (inguinal lymph nodes, upper 2 rows) and muscle at injection site (lower 2 rows) in cynomolgus monkeys after 4 times repeated injections of ZF2001 vaccine. Inguinal lymph nodes and muscle staining was performed using hematoxylin and eosin (H&E, figures ×20, insets ×400). Black arrow = phagocytes, white arrow = lymphocytes, yellow arrow = eosinophils.

At the end of the dosing phase, the draining lymph nodes (inguinal lymph nodes) in the adjuvant control group and, low and high-dose groups (6/6) showed mild hyperplasia of the lymph follicles and lymphoid sinus. The histopathological examination of injection site muscle showed focal basophilic adjuvant deposition, infiltration of the phagocytes, lymphocytes, plasma cells and eosinophils, and quadricep muscle-fiber atrophy and/or necrosis (Figure 4). Histological changes in the draining lymph nodes and injection sites from low and high vaccine groups were consistent with adjuvant control group, suggesting that these changes are induced by the aluminum-containing adjuvants. After a 2-week recovery period, adjuvant deposition and hyperemia and edema persisted in interstitial at the injection site and lymph nodes response, but this was attenuated to some extent compared to the end of the dosing phase. The chronic inflammation response appeared, including mild interstitial hyperplasia and muscle fiber regeneration, showing a trend of recovery.

### 3.2. Immunogenicity

According to the immunization schedule, serum was collected from the animals and detected for the binding antibody and neutralizing antibody. After two times vaccinations, low-dose and high-dose ZF2001 induced cynomolgus monkeys to produce antigen-specific IgG-binding antibodies up to 10^6^ level, and maintain this high level until the end of the study [15]. After two times vaccinations, low-dose and high-dose ZF2001 induced cynomolgus monkeys to produce neutralizing antibodies against SARS-CoV-2 up to 10^3^ level, and maintain high level until the end of the study [15]. After four times vaccinations, low-dose and high-dose ZF2001 induced cynomolgus monkey spleen lymph cells to produce high level IL-2, IFN-γ, IL-4, indicating that the Th1 and Th2 type cellular immune responses were activated. These data were reported in previous studies, showing that ZF2001 induced humoral and cellular immunology responses in cynomolgus monkeys [15].

**Figure 5:**
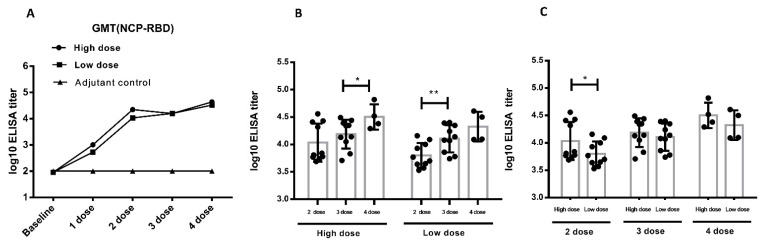
Neutralizing antibody in immunized cynomolgus monkeys based on pseudoviruses (the immunization procedure: 0 W-4 W-8 W-10 W, 4 times, *n* = 10 animals/group, *n* = 4 animals/group during recovery period). (**A**). The level of neutralizing antibody in serum of cynomolgus monkeys during the study (mixed serum). (**B**). A comparison of the neutralizing antibody analysis between different immunization periods in cynomolgus monkeys (individual sera) (**C**). Comparison of the neutralizing antibody analysis between different dose in cynomolgus monkeys (individual sera). * *p* < 0.05, ** *p* < 0.01. Data were presented as Mean ± SD.

The pseudovirus assay was another way to the detect neutralizing antibody approved by Chinese CDC. In addition to the SARS-CoV-2 assay, we used SARS-CoV-2 pseudovirus to detect neutralizing antibody level in vaccinated cynomolgus monkeys. The repeated vaccination induced a strong NTAb response in cynomolgus monkeys. The NTAb level increases to 10^4^ after the second immunization. Antibody titers increased as the vaccination times increased (Figure 5A), suggesting that the vaccine was highly immunogenic in cynomolgus monkeys. In the high-dose group, no significant titer differences were detected between the 2nd and 3rd vaccination, but the 4th vaccination induced significant higher titer than the 3rd vaccination (*p* < 0.05). In the low-dose group, the 3rd vaccination induced significant higher titer than the 2nd vaccination (*p* < 0.01), but titer differences between the 4th and 3rd vaccinations were not detected (Figure 5B). After the 2nd immunization, there was a significantly higher NTAb titer in the high-dose group than in the low-dose group (*p* < 0.05). After the 3rd and 4th immunization, the neutralizing antibody ascended to a certain extent, but there were no significant differences between low-dose and high-dose groups (Figure 5C). As indicated above, both high-dose and low-dose immunization can induce potent neutralizing antibodies, which was consistent with the SARS-CoV-2 NTAb assay. The clinical vaccination frequency should be no less than two doses of ZF2001.

### 3.3. Cardiovascular and Respiratory Safety Pharmacology Study

**Figure 6:**
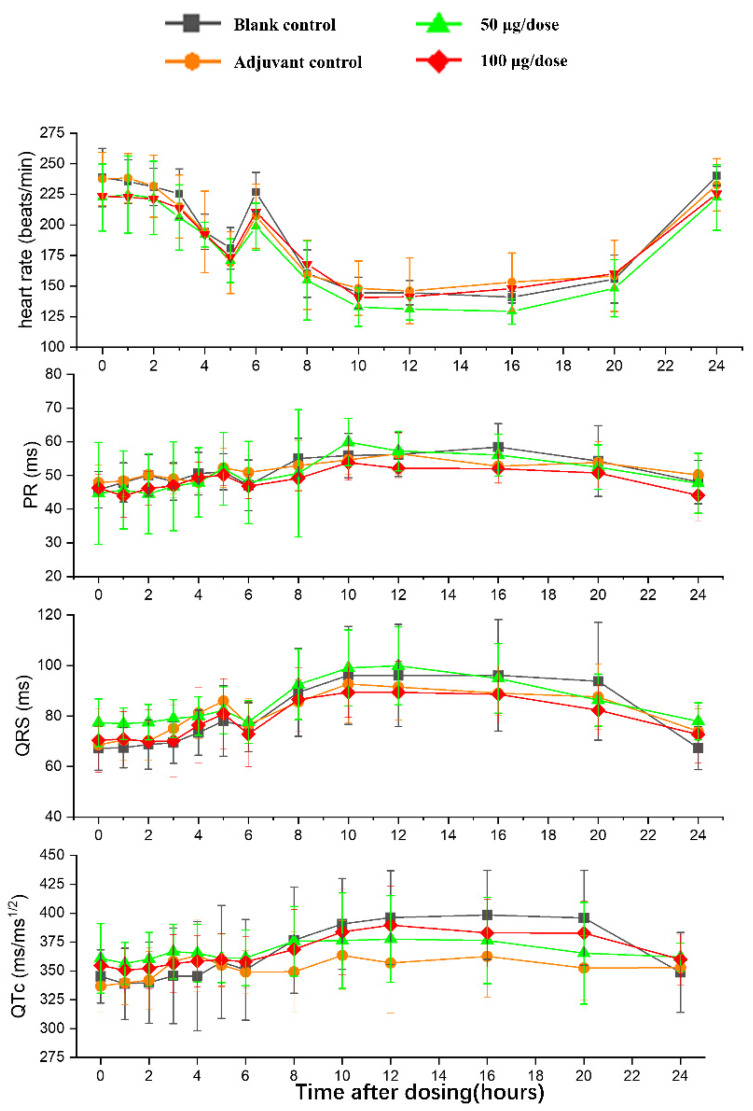
The effects of intramuscular injection of ZF2001 on PR, QRS and QTc in cynomolgus monkeys, *n* = 8 in each group. *p* > 0.05, vs. the blank group.

**Figure 7:**
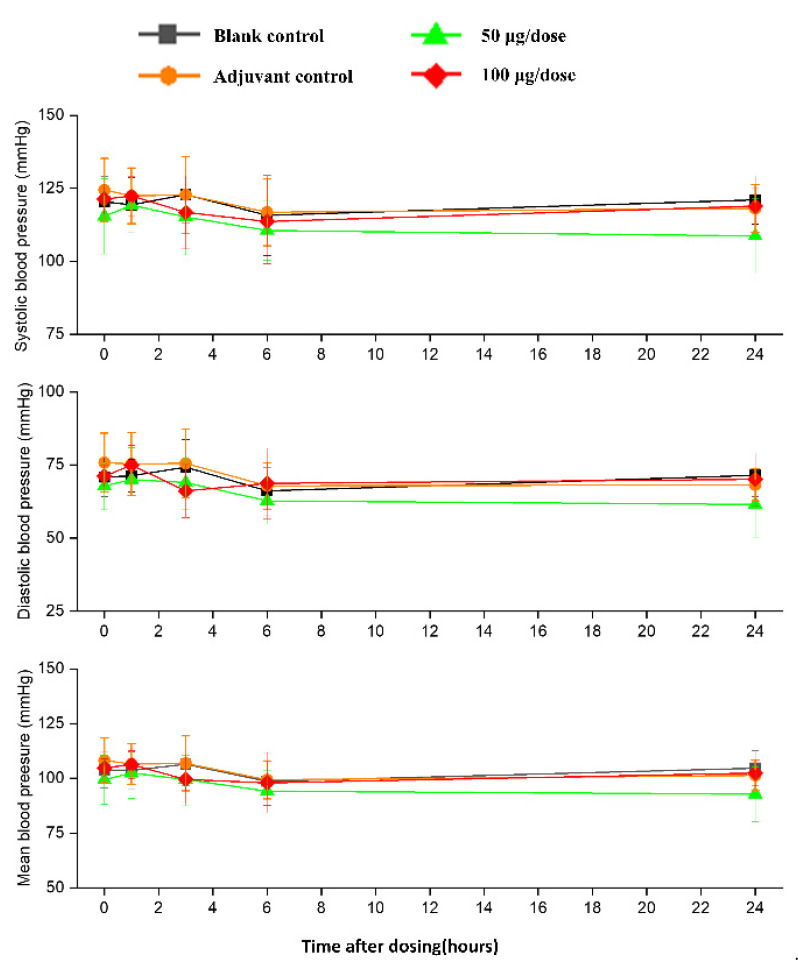
The effects of intramuscular injection of ZF2001 on systolic blood pressure, mean blood pressure and diastolic blood pressure in cynomolgus monkeys, *n* = 8 in each group. *p* > 0.05, vs. the blank group.

**Figure 8:**
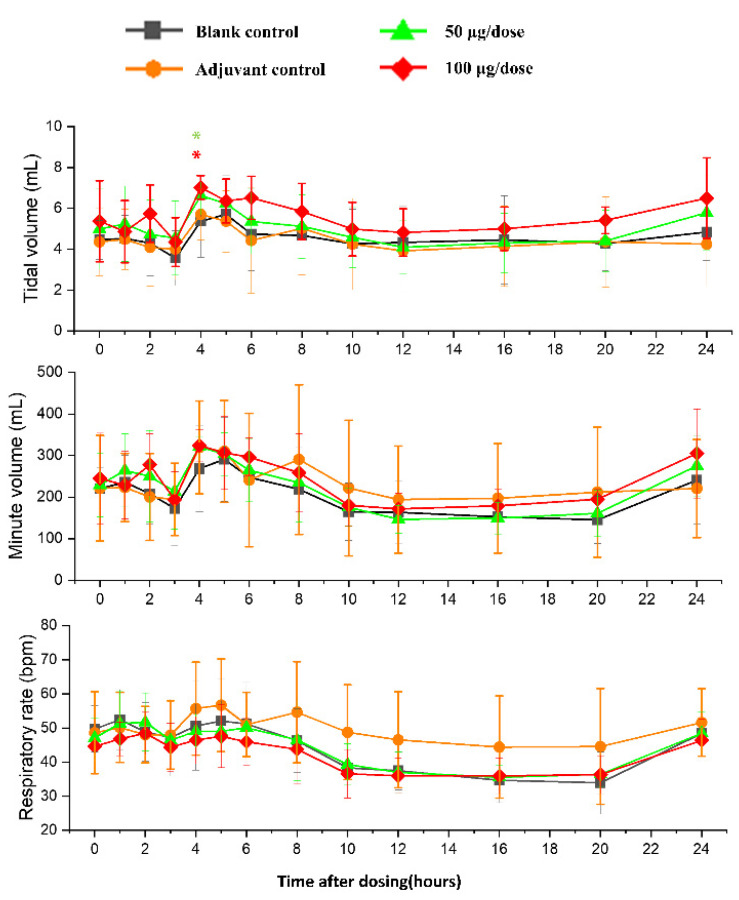
The effects of intramuscular injection of ZF2001 on tidal volume, minute volume and respiration rate in cynomolgus monkeys, * *p* < 0.05 at 3–4 h, *n* = 8 in each group. vs. the blank group.

There was no significant difference in heart rates, PR, QRS, or QTc in the ZF2001 low and high-dose groups compared to the blank group (Figure 6). The significant decrease in ST wave 2–3 h or 16 h after administration in low-dose and high-dose were occurred compared to blank group. No obvious effects of ZF2001 on other indices of ECG were found in cynomolgus monkeys (Table S5). The first administration of ZF2001 at doses of 50 μg and 100 μg resulted in there being no treatment-related effects on any hemodynamic parameters (systolic blood pressure, diastolic blood pressure and mean blood pressure) and body temperature (Figure 7, Table S6). A significant increase in tidal volume 3–4 h after administration was noted in the low-dose and high-dose group compared to the blank group (*p* < 0.05) (Figure 8). However, the above changes were very sporadic and small, and were therefore considered incidental and unrelated to the ZF2001 vaccine.

The changes in ECG, blood pressure, respiration and body temperature up to 24 h after the first dosing were very slight and consistent among all four groups, so these were considered to be unrelated to the treatment. We assumed that they were caused by circadian variations or external human activity.

In brief, the repeated-intramuscular injections of ZF2001 (0 W, 4 W, 8 W, and 10 W, four times in total, following a two-week recovery period) in healthy cynomolgus monkeys induced a series of changes, as follows: adjuvant depositions at the injection site, inflammatory reactions at muscle vaccine-injection sites, hyperplasia of lymph follicles and lymphoid sinuses of local lymph nodes, an increase in blood neutrophils, and a decrease in lymphocyte. The above changes may be related to the inoculation of the aluminum-containing adjuvant vaccine. A single-dose injection of ZF2001 did not lead to a significant respiratory and cardiovascular response in cynomolgus monkeys. No obvious general toxicity and immunity toxicity effects were found. The toxic target organ was not found. The ZF2001 vaccine induced strong immunogenicity against SARS-CoV-2 in this preclinical study. The no observed adverse effect level (NOAEL) in cynomolgus monkeys of ZF2001 vaccine was 100 μg/dose.

## 4. Discussion

In order to prevent the spread of the COVID-19 virus worldwide, a safe and effective vaccine is urgently needed. Prophylactic vaccines are given to the large-scale, healthy population. Therefore, it is essential to ensure the highest safety profile.

Monkeys are a suitable species for biological vaccine research, due to their similar immune response properties to humans and detailed background data [16,17]. In order to provide more detailed toxicological information, we performed a ZF2001 vaccine repeat-dose toxicity study in cynomolgus monkeys after the toxicological study in rats. The SARS-CoV-2 virus causes monkeys to suffer from acute respiratory distress syndrome, which is similar to the effect in humans. A repeat-toxicity study is the key test to evaluate the preclinical safety of vaccines before their entry into clinical trials. It provides systematic animal data to predict potential adverse responses in humans, including the type, extent and reversibility of the adverse responses. Due to the scarcity of experimental monkey resources, we combined safety pharmacology, immunotoxicity and local irritation studies into the repeat-toxicity test through a reasonable design to reduce monkey resources and time costs [18].

In the present study, the ZF2001 vaccination induced a strong humoral immune response against SARS-CoV-2. A high titer of neutralizing antibody and time-dependent neutralizing antibody responses were observed in cynomolgus monkeys. Increasing the number of vaccinations can increase the antibody level to a certain extent. In addition, it is reported that ZF2001 activated the cellular immune response. Splenic lymphocyte isolated from cynomolgus monkey was stimulated by the specific COVID-2019 antigen. This induced a high level of IL-12, IFN-γ, and IL-4, suggesting that the Th1 and Th2 immune response was enhanced [15]. Therefore, ZF2001 can induce humoral and cellular immune responses in cynomolgus monkeys. These findings show that ZF2001 is a promising vaccine for human COVID-2019.

Vaccines elicit the immune organs to induce strong humoral immune responses and cellular immune responses [19,20]. The potential toxicity risk of vaccines often comes from their interference with the immune system [21]. The ZF2001 vaccine induced neither spleen or thymus organ weight/ratio changes, nor histological changes, except local lymph nodes’ hyperplasia and an immune response in the injection site muscle. These occurred equivalently between the adjuvant control and vaccine-treated group and were considered to be caused by an aluminum-containing adjuvant. This was relieved to some extent after a 2-week recovery period. Overall, no obvious immunotoxicity was detected in our study. Four vaccinations, one more dosing than the three clinically planned inoculations, produced no systemic toxicity caused by the repeated administration of ZF2001 regarding weight, temperature, hematology, clinical chemistry, ECG, pathology, etc. Only fluctuation changes were seen to deviate from the baseline of cardiovascular and respiratory parameters in safety pharmacology research. These changes are the same for each group, and are probably caused by the animals’ circadian physiological rhythm.

The antibody-dependent enhancement (ADE) and vaccine enhancement disease (VED) risks caused by SARS-CoV-2 vaccines have been paid more attention in safety evaluations. In animals immunized by the respiratory syncytial virus (RSV), severe acute respiratory syndrome coronavirus (SARS) and Middle East Respiratory Syndrome Coronavirus (MERS) vaccines, the RSV, SARS and MERS challenge can induce pulmonary immunopathological response as eosinophil infiltration which is T helper cells 2 (Th2) highly sensitive [22,23,24]. The respiratory syncytial virus (RSV) caused VED in children who had been immunized by the RSV vaccine. Two cases of death were reported in children, and the pulmonary pathological observation showed extensive monocyte infiltration, mainly consisting of eosinophil [23]. Therefore, the vaccine against SARS-CoV-2, which is also an RNA reverse transcription virus, may cause ADE and VED. Th2-type cell hypersensitivity response, Th1/Th2 type cell immune response imbalance, and eosinophil overactivation maybe the reason for ADE and VED. Vaccines that induce balanced Th1/Th2 cellular immune responses would have better immunogenicity and safety [25,26,27]. In our repeat-toxicity study, we tested the cellular immunity response and its degree. The increase in neutrophils and EOS cells, and the decrease in lymphocytes (proportion and number) in monkey peripheral blood may suggest the activation of cellular immune function. IL4, IL2 and IFN-γ secretion in spleen cells isolated from cynomolgus monkeys were similarly increased, which means that both Th1(IL2, IFN-γ) and Th2 (IL4) type cells immune were activated. The immune balance of Th1/Th2-type cells was maintained. Although we did not detect serum Th1/Th2 type cytokine profiling, which will be helpful to reduce security concerns about ADE or VED, the immunotoxicity and systemic toxicity results in this study shows no abnormal changes, including white cells, IgG, IgM, C3, C4, CD3+, CD4+, CD8+ subtype lymphocyte, pathologic changes in immune organ, respiratory and cardiovascular response, etc. Therefore, ZF2001 may not increase the risk of ADE and VED in cynomolgus monkeys. In the further clinical trials (NCT04445194, NCT04466085 and NCT04646590), ZF2001 has been proven the risk of ADE and VED was relatively low [10,28].

This preclinical study provided the dataset used to obtain approval for clinical trials and the later emergency use of the ZF2001 vaccine in China, Uzbekistan, Indonesia, and Columbia, with more than 200 million doses being administered. Phase I, phase II and phase III clinical trials have shown that the ZF2001 vaccine has good tolerability and potent immunogenicity, and induces long-term protection against SARS-CoV-2 in humans [10,28], which is consistent with our present preclinical study.

## 5. Conclusions

A preclinical safety evaluation of ZF2001 in cynomolgus monkeys shows good tolerance, no general toxicity and no immunotoxicity. These findings have successfully supported the implementation of large-scale clinical trials of the ZF2001 vaccine.

## Figures and Tables

**Table 1:** The hematology analysis in cynomolgus macaques treated with ZF2001 vaccines in repeat-dose toxicity study.

Parameter	End of the Dosing Phase (*n* = 10)				End of the Recovery Period (*n* = 4)			
	Blank Control	Adjuvant Control	Low Dose	High Dose	Blank Control	Adjuvant Control	Low Dose	High Dose
WBC (10^3^/μL)	11.22 ± 2.6	11.18 ± 4.55	11.82 ± 3.5	14.04 ± 4.44	10.16 ± 2.14	9.3 ± 2.08	5.71 ± 1.23	10.51 ± 4.77
RBC (10^6^/μL)	5.55 ± 0.39	5.43 ± 0.27	5.45 ± 0.35	5.56 ± 0.39	6.07 ± 0.45	5.72 ± 0.34	5.83 ± 0.25	6.04 ± 0.47
HGB (g/dL)	134 ± 9	133 ± 7	131 ± 9	132 ± 9	146 ± 8	143 ± 6	141 ± 9	143 ± 6
HCT (%)	42 ± 2.3	42.1 ± 1.8	41.8 ± 2.7	42.1 ± 2.8	45.3 ± 2.3	44.6 ± 1.8	43.5 ± 2.8	45.1 ± 2.3
MCV (fL)	75.8 ± 2.5	77.7 ± 2.7	76.6 ± 2.1	75.8 ± 4.7	74.7 ± 3.2	78.1 ± 1.7	74.5 ± 1.7	74.9 ± 5.5
MCH (pg)	24.1 ± 1.2	24.6 ± 1	24 ± 0.9	23.7 ± 1.2	24.1 ± 1.1	25.1 ± 0.8	24.2 ± 0.5	23.8 ± 1.5
MCHC (g/dL)	318 ± 11	316 ± 8	314 ± 9	313 ± 11	323 ± 2	322 ± 5	324 ± 2	318 ± 5
RDW (%)	12.5 ± 0.5	12.3 ± 0.5	12.4 ± 0.6	12.7 ± 0.8	12.5 ± 0.4	12.7 ± 0.1	12.6 ± 0.5	12.8 ± 0.4
PLT (10^3^/μL)	434 ± 82	434 ± 64	420 ± 54	414 ± 73	479 ± 180	437 ± 59	497 ± 81	441 ± 43
MPV (fL)	8.3 ± 0.8	7.9 ± 0.5	7.6 ± 0.6	7.9 ± 0.9	8.1 ± 1.1	7.5 ± 0.3	7.4 ± 0.4	7.7 ± 0.9
#NEUT (10^3^/μL)	4.08 ± 1.57	6.24 ± 3.58	7.73 ± 2.78 **	8.35 ± 2.92 **	4.65 ± 1.45	4.23 ± 1.76	2.82 ± 1.11	6.68 ± 3.65
#LYMPH (10^3^/μL)	6.47 ± 2.47	4.3 ± 1.83 *	3.28 ± 0.88 **	4.49 ± 1.76 *	5.01 ± 2.44	4.59 ± 1.4	2.51 ± 0.43	3.39 ± 1.02
#MONO (10^3^/μL)	0.34 ± 0.11	0.34 ± 0.17	0.3 ± 0.09	0.37 ± 0.14	0.27 ± 0.09	0.24 ± 0.09	0.2 ± 0.04	0.27 ± 0.14
#EOS (10^3^/μL)	0.19 ± 0.06	0.19 ± 0.16	0.43 ± 0.28	0.7 ± 0.33 **▲	0.13 ± 0.1	0.14 ± 0.06	0.13 ± 0.08	0.1 ± 0.03
#BASO (10^3^/μL)	0.04 ± 0.01	0.03 ± 0.02	0.03 ± 0.02	0.04 ± 0.02	0.04 ± 0.01	0.03 ± 0.01	0.02 ± 0.01	0.03 ± 0.01
#LUC (10^3^/μL)	0.12 ± 0.06	0.09 ± 0.03	0.07 ± 0.03	0.09 ± 0.05	0.07 ± 0.04	0.09 ± 0.03	0.04 ± 0.02	0.05 ± 0.02
#RETIC (10^9^/L)	52.9 ± 7.6	50.7 ± 15.6	50.1 ± 16.1	48.5 ± 12.7	50.8 ± 17.1	47 ± 11.1	63.5 ± 16.7	63.7 ± 11.2
%NEUT (%)	36.9 ± 13.9	54.5 ± 13.0 *	64.3 ± 6.4 **	59.5 ± 6.8 **	47 ± 16.5	44.7 ± 12.7	47.9 ± 9.3	60.1 ± 11.9
%LYMPH (%)	57.1 ± 14.1	39.7 ± 12.0 *	28.2 ± 3.8 **	31.8 ± 5.7 **	48.1 ± 16	50.2 ± 12.6	44.8 ± 6.9	35.4 ± 11.1
%MONO (%)	3 ± 0.7	3 ± 0.9	2.7 ± 1.2	2.6 ± 0.6	2.7 ± 0.8	2.5 ± 0.6	3.8 ± 1.7	2.4 ± 0.4
%EOS (%)	1.7 ± 0.3	1.8 ± 1.7	4 ± 2.9	5.1 ± 2.1 **▲	1.2 ± 0.7	1.4 ± 0.4	2.6 ± 2.4	1.2 ± 0.8
%BASO (%)	0.3 ± 0.1	0.2 ± 0.1	0.2 ± 0.1	0.3 ± 0.1	0.3 ± 0.1	0.3 ± 0.1	0.3 ± 0.1	0.4 ± 0.2
%LUC (%)	1 ± 0.4	0.8 ± 0.3	0.6 ± 0.2 **	0.7 ± 0.3 *	0.7 ± 0.2	0.9 ± 0.2	0.6 ± 0.2	0.6 ± 0.3
%RETIC (%)	0.96 ± 0.15	0.94 ± 0.3	0.92 ± 0.3	0.88 ± 0.25	0.84 ± 0.29	0.83 ± 0.21	1.09 ± 0.3	1.06 ± 0.16

Note: The data were expressed as means ± SD. *, ** Indicate a statistically significant difference at *p* < 0.05 and *p* < 0.01 when compared to the blank control group; ▲ Indicate a statistically significant difference at *p* < 0.05 when compared to the adjuvant control group. Abbreviation: WBC, white blood cell; RBC, red blood cell count; HGB, hemoglobin concentration; HCT, hematocrit; MCV, mean cell volume; MCH, mean cell hemoglobin; MCHC, mean cell hemoglobin concentration; RDW, Red blood Cell distribution width; PLT, platelets; MPV, Mean platelet volume; NEUT, neutrophiles; LYMPH, lymphocytes; MONO, monocytes; EOS, eosinophils; BASO, basophiles; LUC, large unstained cells; RETIC, reticulocyte; PT, Prothrombin time; Fbg, fibrinogen; APTT, activated partial thromboplastin time.

**Table 2:** The blood biochemistry analysis in cynomolgus macaques treated with ZF001 vaccines in repeat-dose toxicity study.

Parameter	End of the Dosing Phase (*n* = 10)				End of the Recovery Period (*n* = 4)			
	Blank Control	Adjuvant Control	Low Dose	High Dose	Blank Control	Adjuvant Control	Low Dose	High Dose
ALT (IU/L)	42.14 ± 8.94	43.42 ± 8.18	48.12 ± 18.22	44.09 ± 17.81	39.66 ± 7.77	43.19 ± 5.52	39.88 ± 5.61	47.42 ± 11.07
AST (IU/L)	42.6 ± 6.46	38.98 ± 7.52	42.67 ± 9.62	38.81 ± 6.29	39.43 ± 12.57	33.26 ± 9.58	36.32 ± 10.25	40.91 ± 3.33
T.P (g/L)	77.33 ± 3.78	79.24 ± 4.21	78.29 ± 4.42	79.22 ± 6.24	81.99 ± 4.15	79.9 ± 4.59	83.81 ± 2.21	85.19 ± 9.4
ALB (g/L)	38.9 ± 9.56	42.52 ± 1.9	43.01 ± 1.78	42.34 ± 3.13	43.7 ± 1.4	43.25 ± 1.35	44.64 ± 1.08	46.4 ± 0.27 **▲
T.BIL (μmol/L)	2.141 ± 0.789	2.518 ± 1.277	2.262 ± 0.521	2.391 ± 1.649	2.523 ± 0.568	3.734 ± 0.739	4.359 ± 1.563	3.684 ± 1.088
ALP (IU/L)	444.01 ± 96.67	428.1 ± 153.06	406.78 ± 161.14	425.4 ± 105.66	553.38 ± 199.43	546.74 ± 220.32	471.71 ± 190.95	481.93 ± 84.43
r-GT (IU/L)	55.03 ± 12.24	65.3 ± 13.77	61.99 ± 15.33	66.14 ± 17.45	60.69 ± 14.13	66.61 ± 12.63	60.89 ± 9.31	71.25 ± 13.3
GLU (mmol/L)	4.873 ± 1.958	4.128 ± 1.674	3.903 ± 1.326	4.669 ± 1.253	3.775 ± 1.018	3.967 ± 0.573	3.54 ± 0.423	3.073 ± 0.422
BUN (mmol/L)	6.458 ± 0.921	6.626 ± 0.845	7.436 ± 1.152	6.487 ± 0.903	6.552 ± 0.904	6.854 ± 0.827	7.782 ± 1.077	7.801 ± 0.523
Crea (μmol/L)	84.43 ± 7.53	88.57 ± 6.95	85.88 ± 9.25	89.76 ± 15.81	82.01 ± 3.03	87.81 ± 8.31	88.43 ± 7.78	99.29 ± 11.57
T.CHO (mmol/L)	3.572 ± 0.679	3.335 ± 0.693	3.369 ± 0.715	3.588 ± 0.858	4.222 ± 0.319	3.264 ± 0.998	4.149 ± 0.497	3.897 ± 1.382
TG (mmol/L)	0.264 ± 0.12	0.32 ± 0.099	0.42 ± 0.159 **	0.364 ± 0.056	0.367 ± 0.181	0.345 ± 0.13	0.398 ± 0.078	0.384 ± 0.039
CK (IU/L)	270.6 ± 56.1	234.2 ± 87.4	223.5 ± 39	280.7 ± 85.1	231.4 ± 77.3	226 ± 47.8	213.1 ± 24.8	246.2 ± 59.9
GLO (g/L)	38.43 ± 12.56	36.72 ± 3.67	35.28 ± 2.87	36.88 ± 4.28	38.29 ± 4.02	36.66 ± 3.48	39.17 ± 3.23	38.79 ± 9.53
A/G	1.12 ± 0.37	1.17 ± 0.12	1.22 ± 0.07	1.16 ± 0.13	1.15 ± 0.13	1.19 ± 0.09	1.15 ± 0.12	1.27 ± 0.4
K^+^ (mmol/L)	4.27 ± 0.69	3.96 ± 0.4	3.81 ± 0.31	3.94 ± 0.43	3.89 ± 0.48	3.77 ± 0.38	3.66 ± 0.16	3.67 ± 0.25
Na^+^ (mmol/L)	149 ± 2	150 ± 2	150 ± 2	151 ± 2	148 ± 1	147 ± 2	148 ± 1	148 ± 1
Cl^−^ (mmol/L)	100.8 ± 1.7	100.5 ± 1.5	101.5 ± 1.4	100.8 ± 2.2	104.6 ± 2.4	104.8 ± 1.1	103.7 ± 0.8	102.1 ± 2.4
Ca (mmol/L)	2.53 ± 0.06	2.54 ± 0.05	2.59 ± 0.09	2.58 ± 0.13	2.59 ± 0.1	2.57 ± 0.06	2.66 ± 0.06	2.63 ± 0.06

Note: The data were expressed as means ± SD. ** Indicate a statistically significant difference at *p* < 0.01 when compared to the blank control group; ▲ indicate a statistically significant difference at *p* < 0.05 when compared to the Adjuvant control group. Abbreviation: ALT, alanine aminotransferase; AST, aspartate aminotransferase; TP, total protein; ALB, albumin; TBIL, total bilirubin; ALP, alkaline phosphatase; r-GT, r-Glutamyltransferase; GLU, glucose; BUN, Blood urea nitrogen; Crea, Creatinine; CHO, cholesterol; TG, triglyceride; CK, creatine phosphokinase; GLO, Globulin; A/G, albumin/globulin ratio.

**Table 3:** The complement C3, C4 and percentage of CD3+, CD4+, CD8+ lymphocytes in cynomolgus macaques treated with ZF001 vaccines in repeat-dose toxicity study.

Parameter	End of the Dosing Phase (*n* = 10)				End of the Recovery Period (*n* = 4)			
	Blank Control	Adjuvant Control	Low Dose	High Dose	Blank Control	Adjuvant Control	Low Dose	High Dose
IgG (g/L)	10.21 ± 3.40	10.06 ± 2.51	9.71 ± 1.77	11.05 ± 2.65	11.2 ± 1.80	10.14 ± 3.02	10.93 ± 2.53	12.44 ± 2.14
IgM (g/L)	0.75 ± 0.36	0.98 ± 0.51	0.80 ± 0.27	0.72 ± 0.38	0.78 ± 0.30	1.04 ± 0.65	0.89 ± 0.22	1.03 ± 0.64
C3 (mg/dL)	108.07 ± 40.73	123.79 ± 22.48	111.18 ± 20.32	114.23 ± 26.51	133.72 ± 26.78	104.76 ± 15.68	126.78 ± 16.92	146.48 ± 21.72
C4 (mg/dL)	21.97 ± 7.92	26.54 ± 8.90	22.78 ± 5.00	23.48 ± 10.27	28.67 ± 3.12	26.42 ± 12.18	29.27 ± 12.53	31.15 ± 13.75
CD3^+^ (%)	60.77 ± 8.70	60.47 ± 7.33	57.32 ± 10.50	57.42 ± 5.16	62.43 ± 4.57	58.62 ± 10.76	51.19 ± 4.73	52.66 ± 5.44
CD3^+^CD4^+^ (%)	58.58 ± 4.63	55.57 ± 5.94	57.81 ± 6.46	58.71 ± 7.67	59.42 ± 5.69	53.47 ± 3.23	53.82 ± 9.88	60.20 ± 6.87
CD3^+^CD8^+^ (%)	33.84 ± 3.70	36.39 ± 4.75	33.83 ± 5.59	32.25 ± 7.7	33.97 ± 4.82	38.22 ± 3.11	38.48 ± 8.15	30.30 ± 4.29
CD4^+^/CD8^+^	1.77 ± 0.33	1.57 ± 0.34	1.78 ± 0.50	1.97 ± 0.75	1.79 ± 0.40	1.40 ± 0.14	1.51 ± 0.69	2.05 ± 0.56

Note: The data were expressed as means ± SD. *p* > 0.05 when compared to the blank control group.

## Data Availability

The data that support the findings of this study are available from the corresponding author [Zhang, L.], upon reasonable request.

## Supplementary Materials

The following supporting information can be downloaded at: https://www.mdpi.com/article/10.3390/vaccines10122080/s1, Table S1: Body weight in cynomolgus macaques treated with ZF001 vaccines in repeat-dose toxicity study. Table S2: Temperature analysis in cynomolgus macaques treated with ZF001 vaccines in repeat-dose toxicity study. Table S3: ECG analysis in cynomolgus macaques treated with ZF001 vaccines in repeat-dose toxicity study. Table S4: Organ weights and organ/body weight ratios in cynomolgus macaques treated with ZF001 vaccines in repeat-dose toxicity study. Table S5: ECG analysis in cynomolgus macaques treated with ZF001 vaccines in safety pharmacology (*n* = 8). Table S6: Body temperature in cynomolgus macaques treated with ZF001 vaccines in safety pharmacology (*n* = 8).
